# Tickborne Relapsing Fever Diagnosis Obscured by Malaria, Togo

**DOI:** 10.3201/eid1301.060670

**Published:** 2007-01

**Authors:** Annika Nordstrand, Ignas Bunikis, Christer Larsson, Kodjo Tsogbe, Tom G. Schwan, Mikael Nilsson, Sven Bergström

**Affiliations:** * Umeå University, Umeå, Sweden; † Association Protestante des Oeuvres Médico-Sociales et Humanitaires du Togo, Lomé, Togo; ‡ National Institutes of Health, Hamilton, Montana, USA; § The Swedish Institute for Infectious Disease Control, Solna, Sweden

**Keywords:** Malaria, Borrelia, public health, West Africa, diagnostics, antimicrobial drug treatment, research

## Abstract

Given the prevalence of relapsing fever (RF) in Senegal, this disease may cause illness and death in other areas of West Africa. We performed a cross-sectional, clinic-based study to investigate the presence of RF in Togo during 2002–2004. Blood samples from patients with fever were examined for RF spirochetes by microscopy, PCR, and DNA sequencing of amplicons and for antibodies to the glycerophosphodiester phosphodiesterase antigen. Although no spirochetes were seen in blood smears, ≈10% of the patients were positive by PCR and ≈13% were seropositive for spirochetes. DNA sequencing demonstrated that *Borrelia crocidurae* and *B. duttonii* were present. Most patients were treated for malaria whether or not plasmodia were observed. Thus, many RF patients originally had a misdiagnosis of malaria, which resulted in ineffective treatment. The inability of microscopic analysis to detect spirochetes compared with PCR demonstrates the need for tests with greater sensitivity.

## Abstract 2

Relapsing fever caused by *Borrelia crocidurae* and *B*. *duttonii* in Togo may be misdiagnosed.

Spirochetes of the genus *Borrelia* are known to cause 2 major types of human disease, Lyme disease, which occurs primarily in temperate regions, and relapsing fever (RF), which occurs in both temperate and tropical regions. Many vertebrates serve as enzootic hosts for the bacteria, and borreliosis is related to climatic and other environmental parameters required for the vectors and reservoir hosts (*1*,*2*). *Borrelia*-related disease is endemic in tropical and subtropical regions; *B*. *hermsii* and *B*. *turicatae* cause tickborne RF (TBRF) in North America. In Europe, TBRF is uncommon; *B*. *hispanica* is the causative agent in Spain, Portugal, Greece, and Cyprus (*3*,*4*).

TBRF in Africa is caused primarily by *B*. *duttonii*, which is transmitted by *Ornithodoros moubata* ticks in East and Central Africa, and by *B*. *crocidurae*, which is transmitted by *O*. *sonrai* in West Africa. Humans are the only known vertebrate host for *B*. *duttonii*. *B*. *crocidurae* is maintained in enzootic cycles in rodents and other small mammals. African TBRF is associated with proximity to tick-infested burrows and huts (*4*–*6*).

The primary clinical manifestations of RF are recurrent high fever interrupted by afebrile periods, hepatomegaly, splenomegaly, and anemia. These signs are similar to those of malaria. The fever peaks are associated with high spirochetemias, and antigenic variation leads to new antigenic variants of a major surface protein and the recurrence of high numbers of borreliae in the blood (*7*–*10*). When patients have high fever, spirochetes may achieve sufficiently high cell densities in the blood to be observed directly by microscopy when wet mounts of blood or Giemsa-stained blood smears are examined. Between peaks, the bacteria are too scarce to be visualized in the blood. Treatment with various antimicrobial drugs is effective (*5*,*6*); however, borreliae may rapidly invade the brain, and infection of the central nervous system may persist if not treated or if treated with antimicrobial drugs that do not readily penetrate the blood-brain barrier (*5*).

Research on RF in Africa has been limited, and little is known regarding the presence and geographic distribution of the spirochetes and tick vectors. Studies in Senegal indicate that RF is widely distributed and prevalent in this country; investigators speculate that RF may cause illness in rural areas throughout much of West Africa (*11*). A 2-year prospective investigation in a rural community in the Senegalese savanna showed that 10% of the study population became infected during the study period, resulting in an incidence of 5.1% (*12*). A recent 14-year longitudinal study demonstrated an average TBRF incidence of 11/100 person-years in Delmo, Senegal, and suggested that TBRF is a common cause of fever in most rural areas of Senegal as well as in some regions of Mauritania and Mali (*13*). In other areas of West Africa, where RF has not been identified, the disease is generally not considered in the diagnosis when patients have a fever.

We hypothesized that RF caused by *B*. *crocidurae* may be present in other areas of West Africa where the climate and environment are similar to that of Senegal. However, because of the lack of knowledge, diagnostics, and the high prevalence of malaria in these areas, RF remains undetected (*14*). Therefore, we conducted a study in Togo to determine if patients with fever might have RF. The study included examination of blood by direct microscopy, molecular methods, and serologic analysis.

## Methods

### Setting

Clinics participating in the study were located in the northern dry savannah and the southern tropical high plateau of Togo. The clinics were at the children’s hospital, Hopital d’Enfants, in Dapaong (urban/semiurban) in northern Togo and rural clinics in southern Togo, including the Centre Medico-Social de Sodo in Sodo, Hopital Bethesda, Agou Clinic in the Agou area, and the general hospital in Kpalimé, a town with ≈50,000 inhabitants (Figure).

**Figure:**
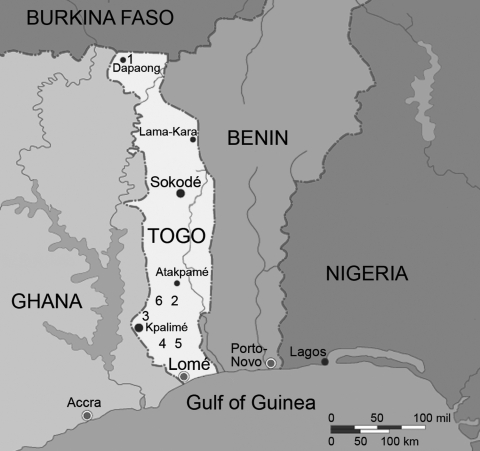
Locations of clinics in Togo involved in the study: 1, Dapaong; 2, Sodo; 3, Kpalimé; 4, Agou; 5, Bethesda; 6, sites of the community study in the Sodo region (adapted from www.maps.com).

### Participants

Interviews and sampling were conducted from March 2002 through September 2004; sampling was performed from August through October in 2003 and 2004 in northern and southern Togo, respectively. Trained laboratory personnel in various clinics obtained blood samples, conducted interviews, and performed microscopic analyses. A total of 244 persons with fever were randomly selected; 14 persons without fever were included as controls.

### Procedures

A questionnaire that contained information on demography, living conditions such as building materials that may be favorable for nesting ticks and rodents, and occupation was used in interviews. Axillary temperature of each patient with fever was measured. Blood was collected from the arm by venipuncture or from a finger by lancet stick and applied to glass microscope slides. Thick and thin blood smears were stained with Giemsa and analyzed by microscopy at a magnification of 1,000× for plasmodia and at 400× for spirochetes. Microscopic examination for spirochetes was used at the clinics to enable diagnosis on site by the method routinely used in Senegal (*14*,*15*). Approximately 300 fields were examined to detect malaria parasites and borreliae. For malaria diagnosis, samples were examined for trophozoites and gametocytes. Blood and serum samples were stored at −20°C and shipped frozen to Sweden for further testing.

### Plasmid Cloning and Protein Expression

Genomic DNA from *B*. *crocidurae* was used as a template for amplification of the glycerophosphodiester phosphodiesterase (*glpQ*) gene and subsequent sequence analysis (Table 1). The PCR amplification product was digested with *Bam*HI and *Xho*I and cloned into the pET-15b vector (Novagen, Madison, WI, USA) as previously described (*16*). The resulting recombinant plasmid was transformed into the Rosetta strain of *Escherichia coli*. The heterologously produced histidine (His)–tagged GlpQ fusion protein was purified by using Ni-NTA spin columns (Qiagen, Valencia, CA, USA), and the protein concentration was determined with Bradford assay (Bio-Rad Laboratories, Hercules, CA, USA).

**Table 1:** Primers used for cloning, PCR, and DNA sequencing

Primer	Sequence (5′→3′)*	Reference
Br_GlpQ_3_*Bam*HI	GGC**GGATCC**GCTTGACCAGTTGCTCCTCCGC	(*16*)
Br_GlpQ_5_XhoI	GCCG**CTCGAG**GAAAAGAAAATGCAAAAATAAATAAA	(*16*)
GlpQ For	GGTATGCTTATTGGTCTTC	Present study
GlpQ Rev	TTGTATCCTCTTGTAATTG	Present study
GlpQ1	CAAATCACTAAGCCTTAGCGAAAGAT	Present study
GlpQ2	ATCTGTTGGTGCTTCTTCCCAGT	Present study
GlpQ3	CAGGGAAAATTGATAATGCTTGTTGG	Present study
GlpQ4	CTGCTAATGTGAAATCGACGGAATA	Present study
Nested_1_F	AGAGTTTGATCCTGGCTTAG	(*15*)
Nested_1_R	CTTGCATATCCGCCTACTCA	(*15*)
Nested_2_F	GGCTTAGAACTAACGCTGGCA	(*15*)
Nested_2_R	CTGCTGGCACGTAATTAGCC	(*15*)

*Sequences in **boldface** indicate restriction sites for *Bam*H1 and *Xho*I.

### ELISA with Recombinant GlpQ Protein

An ELISA was used to detect anti-GlpQ immunoglobulin G (IgG) antibodies in patient sera and was performed as described by Porcella et al. (*16*). Briefly, His-GlpQ protein was adsorbed onto microtiter well surfaces of Ni-NTA HisSorb plates (Qiagen). Wells were blocked with diluent to inhibit nonspecific binding and washed. Serum samples were tested at a 1:100 dilution by incubating 100 μL/well for 1 h at room temperature. After 3 washes, 100 μL of a 1:2,500 dilution of goat anti-human IgG (heavy and light chains) conjugated to horseradish peroxidase (Kirkegaard and Perry Laboratories, Gaithersburg, MD, USA) was added to each well and incubated for 1 h. After 3 washes, 50% 2,2′-azino-di-(3-ethyl-benzthiazoline sulfonate) substrate was added and incubated for 25 min before analysis at 405 nm with a Multiskan microtiter plate reader (Labsystems, Vantaa, Finland). Each serum sample was tested in triplicate, and the mean absorbance value was determined. Samples were considered positive if their mean absorbance was greater than the mean plus 3 standard deviations of the absorbance of control sera tested at the same dilution. Serum samples from Ethiopian patients with RF were included as positive controls (*16*).

### Blood Screening by Nested PCR

Blood samples that were positive or borderline positive by ELISA were tested by a nested PCR for *Borrelia* DNA. DNA was purified from the blood samples and 4 primers (Table 1) were used for detection of the 16S rRNA gene of borreliae. The first PCR amplified a 584-bp region of the gene with the primers Nested_1_F – Nested_1_R. The second PCR amplified a 498-bp region with the primers Nested_2_F – Nested_2_R. The amplicons obtained from the nested PCRs were sequenced to identify the *Borrelia* species because the primers were not able to amplify DNA from specific species of RF spirochetes.

### PCR and Sequencing

For amplification of the complete coding sequence of *glpQ* gene from *B*. *duttonii,* 2 primers were designed by using *B*. *hermsii* noncoding sequences flanking the *glpQ* gene and 4 primers were designed by using sequences within the gene (Table 1). Nested PCR primers (Table 1) were designed to target the 16S rRNA gene. The PCR product for the *B*. *duttonii glpQ* gene was sequenced, and data were deposited in GenBank (accession no. DQ909058).

### Ethics

The study was approved by the Ethics Committee at Umeå University (Dnr 04–050 M). Informed consent was obtained at clinics from all patients or from the accompanying parent if the patient was a child.

### Statistical Analysis

Proportions were compared with a 2-tailed χ^2^-corrected (Yates) analysis and Fisher exact test. p values <0.05 were considered significant.

## Results

No patients were positive for borreliae by microscopic examination of Giemsa-stained blood smears. Among patients with fever, 9 (10%) of 90 children in northern Togo, and 5 (9.8%) of 51 children and 16 (16.3%) of 98 adults in southern Togo were seropositive by ELISA. A total of 12.6% of patients with fever were positive by ELISA (Table 2). For those patients without fever, 2 (14.3%) of 14 were seropositive. Because the *glpQ* gene is present in RF spirochetes but Lyme disease spirochetes are not, the positive serologic results strongly suggest that patients were infected with RF spirochetes (*11*). However, this serologic test is not species specific and cannot distinguish current from past infections.

**Table 2:** Prevalence and identification of *Borrelia* infections in patients with fever at clinics in northern and southern Togo, 2002–2004

Region, age group, y	Seropositive for relapsing fever *Borrelia*,* no. positive/no. tested (%)	*Borrelia* in blood,† no. positive/no. tested (%)	Infected with *B. crocidurae*,‡ no. positive/no. tested (%)	Infected with *B. duttonii*,‡ no. positive/ no. tested (%)
Northern				
0–4	6/60 (10)	4 (6.7)	3 (5)	1 (1.7)
5–14	3/30 (10)	4 (13.3)	3 (10)	1 (3.3)
Total	9/90 (10)	8 (8.8)	6 (6.7)	2 (2.2)
Southern				
0–4	2/16 (12.5)	1 (6.3)	1 (6.3)	0
5–14	3/35 (8.6)	2 (5.7)	2 (5.7)	0
Total	5/51 (9.8)	3 (5.9)	3 (5.9)	0
15–24	7/43 (16.3)	3 (7)	3 (7)	0
>25	9/55 (16.4)	7 (12.7)	7 (12.7)	0
Total for adults	16/98 (16.3)	10 (10.2)	10 (10.2)	0
Total	21/149 (14.1)	13 (8.7)	13 (8.7)	0
All	30/239 (12.6)	21 (8.8)	19 (7.9)	2 (1.2)

*Determined by ELISA for glycerophosphodiester phosphodiesterase antigen. †Determined by 16S rRNA gene PCR amplification from blood of patients with high ELISA values divided by all ELISA- tested patients. ‡Determined by genomic sequence of 16S rRNA in blood samples.

Current *Borrelia* infections were detected by PCR and 16S rRNA gene sequence analysis in blood samples of patients from both northern and southern Togo. DNA sequencing identified both *B*. *crocidurae* and *B. duttonii*, but *B. duttonii* was found only in patients from northern Togo (Table 2). All 81 patients from northern Togo who were seronegative were also negative by PCR. In contrast, 8 (88.9%) of 9 patients who were positive by ELISA were also positive by PCR (p<0.05). All patients who were positive by PCR had a fever when their blood samples were collected.

A total of 28 patients from southern Togo were tested for current spirochetemias and included all ELISA-positive and some ELISA-negative patients. The negative samples chosen were those with the highest values below the cut-off value as well as randomly chosen samples with lower values. The 13 PCR-positive patients included 11 (55%) of 20 ELISA-positive patients and 2 (25%) of 8 ELISA-negative patients. Two samples from ELISA-positive patients could not be tested by PCR because the DNA was degraded. Both patient samples that were PCR positive and ELISA negative had ELISA values just below cut-off value used in the study.

All patients from northern Togo were children (age range <1–14 years). For children <4 years of age, 6 (10%) of 60 were seropositive. Of these children, 5 had a fever and 4 had an active *Borrelia* infection detected by PCR. DNA sequence analysis showed that 3 children were infected with *B*. *crocidurae* and 1 with *B*. *duttonii*. In southern Togo, 1 (9.1%) of 11 children <1–4 years of age were seropositive. The overall male-to-female ratio among study patients was 1.3:1. Serologic and PCR results did not differ by sex, ethnic background, or profession, with the exception of cow herders. The prevalence of seropositive adults was 62.5% (5/8) among cow herders compared with 12.2% (11/90) among those in other professions (p<0.05); 11 (78.6%) of 14 adults in the Peuhl ethnic group were cow herders (95% confidence interval [CI] 49.2%–95.3%), and 4 of 5 ELISA-positive Peuhl were cow herders. A total of 10.8% (95% CI 5.9%–17.8%) of all adults studied were cow herders. More *Borrelia*-infected patients lived in houses made of mud rather than cement than persons without RF infection (p = 0.008, data not shown).

The prevalence of malaria among patients with fever was 63.1%. Of 21 patients with PCR-confirmed *Borrelia* infections, malaria was diagnosed for 7 on the basis of a positive blood smear. Therefore, 7 (4.5%) of 154 patients were coinfected with malaria parasites and *Borrelia* (Table 3). In the youngest children (<4 years of age), 4 of 5 *Borrelia*-infected children also had malaria.

**Table 3:** Coinfection with malaria and relapsing fever caused by *Borrelia* and treatment in patients in northern and southern Togo with fever, 2002–2004

Region, group	Malaria infection,* no. positive/ no. tested (%)		*Borrelia* infected† and treated for malaria, no. positive/no. tested (%)		*Borrelia* infections effectively treated, no. positive/no. tested (%)
	All patients	*Borrelia* infected	Malaria positive	Malaria negative‡	
Northern					
Children	34/96 (35.4)	4/8 (50)	4/4 (100)	1/4 (25)	0/8
Southern					
Children	46/68 (67.6)	2/3 (66.7)	1/2 (50)	0/1	1/3 (33.3)
Adults	35/80 (43.8)	1/10 (10)	0/1	1/6 (16.6)	0/7
Total	81/148 (54.7)	3/13 (23.1)	1/3 (33.3)	1/7 (14.3)	1/10 (10)
All	154/244 (63.1)	7/21 (33.3)	5/7 (71.4)	2/11 (18.2)	1/18 (5.6)

*Determined by microscopy of Giemsa-stained blood smears. †Determined by positive PCR result and *Borrelia* species identification by sequence in blood. Three malaria-negative adults were not included because treatment information was not available. ‡Values represent patients treated only for malaria. Two of these 4 patients were treated for malaria in combination with drugs for treating helminth infections.

In northern Togo, patients infected with malaria and *Borrelia* were treated primarily with chloroquine, artemether, quinine, and amoxicillin. *Borrelia*-infected, malaria-negative patients were treated primarily with chloroquine, quinine, or amoxicillin. In southern Togo, patients with malaria and *Borrelia* infections were treated primarily with quinine and chloramphenicol for typhoid fever, metronidazole for amebiasis, and albendazole for digestive parasitosis. *Borrelia*-infected, malaria-negative patients were treated with chloroquine and antimicrobial drugs, such as amoxicillin, which are ineffective against RF *Borrelia* (Table 3) (*14*).

## Discussion

Although microscopic analysis of Giemsa-stained blood smears for *Borrelia* showed negative results, current infections were demonstrated by PCR and gene sequencing in 8.8% of patients with fever. Therefore, no RF infections would have been detected if only microscopic examination of stained blood smears had been performed. Our results emphasize the low sensitivity of microscopy in the diagnosis of RF, as has demonstrated by others (*11*,*14*,*17*). Another problem in diagnosis is that the borreliae are detectable only during the short peaks of fever (*18*). Microscopy might have shown positive results if dark-field or fluorescent microscopy had been used, but this was not possible in the clinics in this study. However, 1 of the primary objectives of this study was to improve the diagnosis of RF borreliosis by using molecular and serologic techniques.

A study of rodents in Senegal showed that 57.7% of *Borrelia* infections were false negative by microscopy (*11*). Microscopy is the method routinely used to diagnose *B*. *crocidurae* infections in Senegal, where the disease is common (*14*,*15*). Thus, methods that provide greater sensitivity are needed to determine more accurately the presence of RF throughout West Africa. Since the GlpQ antigen is present in RF *Borrelia* but absent in Lyme disease *Borrelia*, positive antibody test results strongly indicated that infections with RF *Borrelia* were occurring or had occurred (*16*). However, the infecting species and the time of infection could not be determined by this method. Positive serologic test results correlated with current infection, as demonstrated by PCR and sequence analysis. Thus, serologic tests may be more adequate for diagnosis, although ELISA procedures would have to be modified for use in small rural clinics.

Our finding of TBRF in Togo demonstrates that the geographic distribution of this disease in West Africa is greater than previously thought. Trape et al. suggested that RF caused by *B*. *crocidurae* might be spreading to new areas because of the sub-Saharan drought, which might allow vector ticks to colonize new areas in the savannas of West Africa (*2*). Our results suggest that this might be true. We also found RF caused by *B*. *crocidurae* in the tropical region of southern Togo, where the climate may be less favorable for its tick vector. Thus, habitats believed to be preferred by *O*. *sonrai* may need to be reconsidered. Southern Togo has been subjected to deforestation, periods of drought, and slash-and-burn agriculture. During the rainy season, the average temperatures in Dapaong and Atakpame are 24°C and 25.8°C, respectively, compared with dry season mean temperatures of 28.9°C and 26.8°C, respectively (*19*). The dry wind or harmattan from the Sahara Desert during winter can cause periodic droughts in northern Togo. We propose that areas with similar climate in West Africa are likely to have TBRF.

Of particular interest was our finding of *B*. *duttonii* in northern Togo, which extends the known distribution of this species in Africa. Additional work is needed to determine if its vector *O*. *moubata* is present in northern Togo or if *B duttonii* can be transmitted by *O*. *sonrai*. More studies are needed to determine the distribution of RF spirochetes and their vectors in Africa and what effect these infections have on human health.

Many of the primary symptoms of malaria and RF are similar, such as recurrent fever, chills, anemia, hepatomegaly, splenomegaly, and possible neurologic symptoms. Thus, a considerable risk for misdiagnosis of TBRF as malaria exists in countries in which RF is not recognized but in which malaria is prevalent. Occasional reports of European tourists returning home from Senegal with *B*. *crocidurae* infections have implied that the disease may be misdiagnosed as malaria, which leads to incorrect treatment (*14*,*15*,*20*). Because RF has not been investigated in most West African countries including Togo, the disease is generally not considered in a differential diagnosis for fever patients. Also, malaria is often diagnosed on the basis of only clinical symptoms, not examination for parasites in the blood, which increases misdiagnosis. Increased knowledge of the geographic distribution and epidemiology of RF in West Africa should improve the recognition and treatment of this disease. Prompt diagnosis of RF would also reduce the number of people in whom chronic symptoms such as neuroborreliosis later develop when bacteria cross the blood-brain barrier and infect the central nervous system (*5*,*21*,*22*).

We used a questionnaire to determine possible risk factors associated with RF. Some children <1–4 years of age were infected with RF *Borrelia*. Since *O*. *sonrai* is nocturnal, feeding mostly at night, these children may have been infected at home. However, RF among infants may also reflect infection from mothers before or during birth, as occurs with *B*. *duttonii* in East Africa (*18*). We also found that persons with current *Borrelia* infections more often lived in houses made of mud rather than cement. This observation is similar to ones in Senegal, where *Ornithodoros* ticks feed primarily at night inside mud or in adjacent areas where small mammals are present (*2*,*6*,*20*).

Before our study, we trapped 66 rodents and insectivores in or near houses in Togo. Four species were identified: musk shrew (*Crocidura* spp.), Nile rat (*Arvicanthis niloticus*), multimammate rat (*Mastomys natalensis*), and brown rat (*Rattus norvegicus*); 3 are known reservoirs for *B*. *crocidurae* in Senegal. These findings showed the presence of these potential reservoirs in northern and southern Togo (unpub. data). We found that mud huts were associated with entrances of rodent burrows, which might increase the risk for exposure to ticks (data not shown). In the present study, cow herders were also at a greater risk of acquiring RF. However, more studies are required to investigate potential risk groups. A higher proportion of cow herders who were ELISA positive were also Peuhl, who are nomadic people. Their behavior and sleeping outside at night may put them at greater risk of being bitten by nocturnal soft ticks.

We report TBRF in Togo with a prevalence of 8.8% among the patients studied. For those with fever, 63.1% had malaria and 4.5% were coinfected with RF *Borrelia*. Among those with RF, 33.3% were coinfected with malaria parasites. Given the retrospective finding of spirochetes by PCR, only 1 of 18 patients with TBRF received treatment effective against this disease at the time she was seen in the clinic. Thus, TBRF is obscured by the high incidence of malaria in Togo, and this problem likely occurs in other regions in West Africa. In rural health centers without laboratory facilities, diagnosis of malaria is based only on clinical symptoms. The potential risk for misdiagnosis and ineffective treatment of patients with RF rather than malaria needs to be addressed. Our findings demonstrate the need for improved diagnostic procedures to detect TBRF in West Africa. We consider the high prevalence of RF among febrile children and the lack of correct treatment as important health concerns, particularly with regard to the severity of untreated neuroborreliosis and women infected during pregnancy.
